# From Evidence to Delivery: Building Readiness for Maternal Respiratory Syncytial Virus Vaccination in Bangladesh

**DOI:** 10.1002/hcs2.70091

**Published:** 2026-07-29

**Authors:** Ridwana Maher Manna, Siobhan L. Johnstone, Pradip Chandra, Michelle J. Groome, Shams El Arifeen, Aniqa Tasnim Hossain

**Affiliations:** ^1^ Maternal and Child Health Division, International Centre for Diarrhoeal Disease Research Bangladesh (icddr,b) Dhaka Bangladesh; ^2^ South African Medical Research Council Vaccines & Infectious Diseases Analytics Research Unit University of the Witwatersrand Johannesburg South Africa

**Keywords:** antenatal care, Bangladesh, maternal immunisation, maternal vaccine readiness, respiratory syncytial virus, The Maternal Immunisation Readiness Network in Africa and Asia

**Abbreviations**
ANC: antenatal care
LMIC: low‐ and middle‐income country
RSV: respiratory syncytial virus

## 1 Evidence From Global and National Sources

Respiratory syncytial virus (RSV) is often regarded as a mild respiratory infection in older children and adults, a perception that does not reflect the high risk it carries for young infants. RSV is a leading cause of acute lower respiratory tract infection, bronchiolitis, and pneumonia in children under 5 years of age [1].

Globally, RSV accounts for a large share of childhood respiratory morbidity and mortality, with the highest burden occurring in low‐ and middle‐income countries (LMICs). A comprehensive analysis published in *Lancet* in 2024 estimated that RSV causes 33 million lower respiratory tract infections, approximately 3.6 million hospitalisations, around 26,300 in‐hospital deaths, and 118,200 total deaths each year among children under 5 years worldwide [1]. More than 95% of these deaths occurred in LMICs, with infants younger than 6 months bearing the highest risk of severe disease and death [2].

In Bangladesh, Global Burden of Disease analyses identify RSV as an important contributor to neonatal and infant mortality, with RSV‐associated pneumonia contributing substantially to deaths among children under 5 years [3]. National surveillance data further support this burden, reporting RSV positivity of approximately 23% among children under 2 years presenting with severe acute respiratory infection [4]. This article discusses emerging global evidence on maternal RSV immunisation and its implications for implementation and readiness in Bangladesh and other LMIC settings.

## 2 Maternal Immunisation as a Preventive Strategy

Maternal immunisation provides a practical and effective way to protect these infants during early life. Vaccination during pregnancy induces maternal antibody responses that are transferred transplacentally to the foetus, providing passive immunity from birth through the first months of life. This approach is well established. In Bangladesh, maternal tetanus toxoid vaccination has been successfully implemented for decades, contributing to the near elimination of neonatal tetanus and highlighting the feasibility of pregnancy‐based immunisation.

Globally, maternal vaccines for influenza, pertussis, and COVID‐19 are now routinely recommended in many settings. The Abrysvo vaccine (Pfizer, New York, United States) was approved in 2023 following a specific development trajectory. In 2023, the first RSV vaccine for use during late pregnancy (32–36 weeks) was approved by the United States Food and Drug Administration; in September 2024, the Strategic Advisory Group of Experts on Immunization made global recommendations to introduce passive immunisation (maternal vaccination and infant monoclonal antibodies) for the prevention of severe RSV disease in young infants; and in 2025 it received prequalification from the World Health Organization [5].

## 3 What the Trials and Early Implementation Studies Show

Recently, phase three trial data using an updated prefusion F protein‐based vaccine (GSK, Rixensart, Belgium) showed vaccine efficacy exceeding 65% against severe RSV disease during early infancy [6].

Real‐world evidence is now emerging. In 2024, Argentina incorporated the RSV prefusion F vaccine into its national immunisation schedule and delivered it through existing antenatal care (ANC) and vaccination services across public and private healthcare sectors [7, 8]. The programme achieved approximately 60% coverage among eligible pregnant women and was associated with a nearly 75% reduction in severe RSV disease among infants, while all RSV‐associated in‐hospital deaths occurred among infants born to unvaccinated mothers [8]. Argentina's experience highlights the importance of provider engagement, maternal awareness, timely ANC attendance, and coordination across public and private healthcare sectors. Although direct comparison with Bangladesh is limited, these lessons may inform future maternal RSV vaccine introduction.

## 4 Readiness Challenges in Bangladesh

Despite achieving high coverage in childhood immunisation under the Expanded Programme on Immunization, Bangladesh does not yet have a dedicated maternal immunisation platform. ANC services are delivered through a mixed system, with a large proportion of women receiving care from private facilities. Given the important role of private‐sector ANC services in Bangladesh, successful maternal RSV vaccine delivery would require coordination across public and private providers.

While maternal tetanus vaccination has been successfully implemented for decades [9], maternal RSV vaccination presents additional challenges because it must be delivered within a specific gestational window [7]. Late ANC initiation, incomplete follow‐up, and variability in ANC quality may affect timely vaccine delivery. Although not directly comparable, antenatal corticosteroid use illustrates the feasibility of delivering time‐sensitive interventions through routine ANC. Successful introduction will require strengthening ANC attendance, provider counselling, public awareness, and coordination across healthcare sectors.

## 5 Building Momentum Through Readiness Initiatives

Bangladesh is a participant in the Maternal Immunisation Readiness Network in Africa and Asia, a nine‐country initiative supporting evidence generation, health system readiness, stakeholder engagement, and economic evaluation for maternal vaccines [10].

In 2025, International Centre for Diarrhoeal Disease Research, Bangladesh and Irimi convened a national stakeholder workshop using the “6As” behavioural framework (namely Accountability, Access, Affordability, Awareness, Acceptance, and Activation) to identify barriers and potential solutions for maternal immunisation uptake. Key priorities included provider engagement, communication strategies, maternal awareness, provider recommendation, and timely ANC attendance as important determinants of maternal RSV vaccine uptake [11].

## 6 Implications for Policy and Practice

Discussions on maternal RSV vaccination have been initiated in Bangladesh. Policymakers may consider its future inclusion in national immunisation guidelines, aligned with World Health Organization recommendations and potential Gavi support. Integration into routine ANC across public and private sectors will require clear delivery pathways, provider training, and effective counselling tools. As Bangladesh transitions within the LMIC context and the global financing landscape evolves, affordability and long‐term financing should remain important policy considerations.

## 7 Looking Forward

Maternal immunisation presents a promising opportunity to enhance protection for infants in early life while fostering stronger connections between maternal and child health services. In Bangladesh, the establishment of a dedicated maternal immunisation platform could support the equitable introduction of future maternal vaccines, though several implementation and health‐system considerations will need to be addressed.

## Author Contributions

**Ridwana Maher Manna:** conceptualisation, investigation, visualisation, writing – original draft. **Siobhan L. Johnstone:** methodology, validation, writing – review and editing. **Pradip Chandra:** resources, validation, writing – review and editing. **Michelle J. Groome:** methodology, validation, writing – review and editing. **Shams El Arifeen:** project administration, validation, supervision, writing – review and editing. **Aniqa Tasnim Hossain:** project administration, methodology, validation, supervision, writing – review and editing.

## Funding

The authors have nothing to report.

## Ethics Statement

The authors have nothing to report.

## Consent

The authors have nothing to report.

## Conflicts of Interest

The authors declare no conflicts of interest.

## Data Availability

Data sharing is not applicable to this article as no new data were created or analysed in this article.
